# Exploring Text Classification Systems for Automatically Coding Historical Occupations and Causes of Death

**DOI:** 10.23889/ijpds.v9i5.2865

**Published:** 2024-09-18

**Authors:** Mike Jarrett, Brent Hills, Maria Kim-Bautista, Megan Ahuja, Jim Mintha, Gitta Oldendorff, Brett Wilmer

**Affiliations:** 1 Population Data BC; 2 BC Ministry of Citizens' Services - BC Stats

## Objective and Approach

Population Data BC (PopData) transitioned to Research IDs from Study IDs for the Data Innovation (DI) Program, with the objective of improving efficiency of data preparation, linkage, and provisioning while maintaining privacy standards. Study IDs are person-specific encrypted identifiers unique to each study, preventing Researchers from combining records from different projects, in turn reducing risk of re-identification. Research IDs are also person-specific encrypted identifiers, except they are not project specific. Advancements in technology and changes in data provisioning models have reduced utility of Study IDs.

## Results

Implementing Research IDs instead of Study IDs decreased: processing, setup/review, per transfer effort, errors/omissions, storage requirements, staff training/knowledge, licenses, record keeping, specialized knowledge, and per project data tracking. There was a marginally higher risk of re-identification with Research IDs, counter-acted by a higher degree of technological security and stronger procedural and ethical frameworks, resulting in the ability to provide more data to more users.

## Conclusions

Adopting Research IDs has allowed PopData and the DI Program to access established data resources faster, more efficiently, with less per project person effort and reduced storage requirements without a net risk change. The benefits of adopting Research IDs, counter a potential minimal risk gain arising from replacement of Study IDs with Research IDs.

## Implications

Efficiency gains from the use of Research IDs allows PopData and the DI Program to redistribute operational and staff resources, in turn increasing their ability to scale, to provide data in more diverse formats and improve service provision.

